# Trends in the incidence of pulmonary nodules in chest computed tomography: 10-year results from two Dutch hospitals

**DOI:** 10.1007/s00330-023-09826-3

**Published:** 2023-06-20

**Authors:** Ward Hendrix, Matthieu Rutten, Nils Hendrix, Bram van Ginneken, Cornelia Schaefer-Prokop, Ernst T. Scholten, Mathias Prokop, Colin Jacobs

**Affiliations:** 1 Radiology Department, Jeroen Bosch Hospital, Henri Dunantstraat 1, 5223 GZ ‘s-Hertogenbosch, the Netherlands; 2 Department of Medical Imaging, Radboud University Medical Center, Geert Grooteplein Zuid 10, 6525 GA Nijmegen, the Netherlands; 3 Jheronimus Academy of Data Science, Sint Janssingel 92, 5211 DA ‘s-Hertogenbosch, the Netherlands; 4 Radiology Department, Meander Medical Center, Maatweg 3, 3813 TZ Amersfoort, the Netherlands; 5 Radiology Department, University Medical Center Groningen, Hanzeplein 1, 9713 GZ Groningen, the Netherlands

**Keywords:** Multiple pulmonary nodules, Incidence, Tomography, X-ray computed, Natural language processing

## Abstract

**Objective:**

To study trends in the incidence of reported pulmonary nodules and stage I lung cancer in chest CT.

**Methods:**

We analyzed the trends in the incidence of detected pulmonary nodules and stage I lung cancer in chest CT scans in the period between 2008 and 2019. Imaging metadata and radiology reports from all chest CT studies were collected from two large Dutch hospitals. A natural language processing algorithm was developed to identify studies with any reported pulmonary nodule.

**Results:**

Between 2008 and 2019, a total of 74,803 patients underwent 166,688 chest CT examinations at both hospitals combined. During this period, the annual number of chest CT scans increased from 9955 scans in 6845 patients in 2008 to 20,476 scans in 13,286 patients in 2019. The proportion of patients in whom nodules (old or new) were reported increased from 38% (2595/6845) in 2008 to 50% (6654/13,286) in 2019. The proportion of patients in whom significant new nodules (≥ 5 mm) were reported increased from 9% (608/6954) in 2010 to 17% (1660/9883) in 2017. The number of patients with new nodules and corresponding stage I lung cancer diagnosis tripled and their proportion doubled, from 0.4% (26/6954) in 2010 to 0.8% (78/9883) in 2017.

**Conclusion:**

The identification of incidental pulmonary nodules in chest CT has steadily increased over the past decade and has been accompanied by more stage I lung cancer diagnoses.

**Clinical relevance statement:**

These findings stress the importance of identifying and efficiently managing incidental pulmonary nodules in routine clinical practice.

**Key Points:**

*• The number of patients who underwent chest CT examinations substantially increased over the past decade, as did the number of patients in whom pulmonary nodules were identified.*

*• The increased use of chest CT and more frequently identified pulmonary nodules were associated with more stage I lung cancer diagnoses.*

**Supplementary Information:**

The online version contains supplementary material available at 10.1007/s00330-023-09826-3.

## Introduction

Randomized controlled trials for lung cancer screening have provided evidence that lung cancer–related mortality can be significantly reduced when the cancer is detected at an early stage and still appears as a nodular lesion [1, 2]. The outcomes of these trials also underline the importance of accurate detection and management of pulmonary nodules outside a screening setting. Hence, the British Thoracic Society (BTS) [3] and the Fleischner Society [4] have established guidelines for the management of incidental nodules to aid radiologists in their decision-making process and to standardize nodule management.

Despite the existence of these guidelines, little data has been published about the incidence of benign and malignant incidental pulmonary nodules in a routine clinical setting, especially in Europe [5]. In the USA, Gould et al [6] performed a large epidemiological study on the trends of reported pulmonary nodules in chest CT from hospitals and medical offices throughout Southern California between 2006 and 2012. They found that the frequency of nodule identification increased from 24 to 31% for all scans performed. However, most remarkably, the incidence of cancerous nodules remained stable regardless.

It is essential to update our knowledge about the incidence of incidental nodules, taking into account the reported increase in nodule identification rate [6], advances in CT scan technology [7], and increased awareness of the risks of nodules due to the outcomes of lung cancer screening trials. Updated statistics are important in clinical decision-making and risk communication between physicians and patients with incidental nodules [8]. In addition, these statistics could aid shaping the research agenda towards finding solutions to cope with the increasing demand for healthcare [9], such as the implementation of artificial intelligence (AI) solutions.

Therefore, the aim of this study was to conduct a large-scale analysis to examine the trends in the incidence of reported pulmonary nodules in chest CT in two large Dutch hospitals in the period of 2008 to 2019. Nodule incidence was correlated with lung cancer diagnosis to assess the clinical relevance of increased nodule detection.

## Materials and methods

### Study design and data collection

**Fig. 1:**
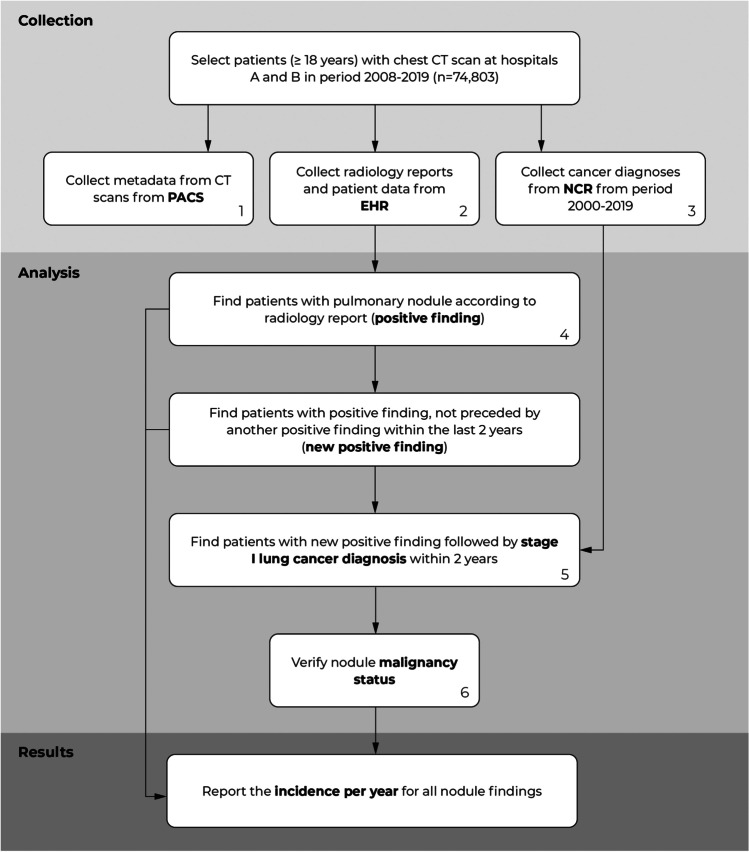
Flow diagram of the data collection and analysis. (1) Netherlands Cancer Registry (NCR). (2) Electronic Health Records (EHR), which stores both radiology reports and patient information (i.e., age, gender). (3) Picture Archiving and Communication System (PACS), which stores all the metadata of the CT scans (i.e., slice thickness, scanner model). (4) Based on a Natural Language Processing (NLP) analysis. (5) Supplementary analysis of other lung cancer stages can be found in Appendix E8 (supplement). (6) The nodule and corresponding lung cancer were manually linked; more details can be found in Appendix E2 (supplement)

We conducted a retrospective study to identify the incidence of pulmonary nodules in chest CT scans in a university medical center (hospital A) and a large peripheral teaching hospital (hospital B) in the Netherlands. For this purpose, we analyzed the radiology reports from 166,688 chest CT studies and the corresponding lung cancer diagnoses in the years 2008 until 2019 (Table 1). The radiology reports were collected from the Electronic Health Record (EHR) systems. Metadata of the CT scans (i.e., slice thickness) were collected from the Picture Archiving and Communication System (PACS). All cancer diagnoses (pulmonary and extrapulmonary) from the period 2000 to 2019 were obtained from the Netherlands Cancer Registry (NCR), managed by the Netherlands Comprehensive Cancer Organization (IKNL). The NCR comprises all individuals diagnosed with cancer in the Netherlands. At both institutions, the institutional review board waived the need for informed consent because of the retrospective design and the use of anonymized data in this study. A flow diagram of the data collection and analysis procedures is shown in Fig. 1.

**Table 1:** Description of the dataset obtained from hospitals A and B, period 2008–2019

		All	Hospital A	Hospital B
No. of patients	All	74,803	40,440	34,363
	Men	40,620	22,581	18,039
	Women	34,183	17,859	16,324
Patient age^1^	All	60.0 ± 16.0	58.1 ± 16.3	62.3 ± 15.4
	Men	60.2 ± 16.0	58.2 ± 16.4	62.7 ± 15.0
	Women	59.9 ± 16.1	58.0 ± 16.1	61.9 ± 15.8
No. of studies		166,688	98,479	68,209
Studies per CT scanner	Siemens Sensation 64	48,203	13,108	35,095
	Siemens SOMATOM Definition Flash	25,695		25,695
	Siemens SOMATOM Definition AS +	3949		3949
	Siemens SOMATOM Definition Edge	1987		1987
	Canon Medical Systems Aquilion One	31,691	31,691	
	Siemens Sensation 16	28,769	28,769	
	Canon Medical Systems Aquilion CXL	10,278	10,278	
	Canon Medical Systems Aquilion Precision	9286	9286	
	Siemens Biograph 40	3126	3126	
	Other^2^	3704	2221	1483

^1^Age at the first examination during the investigated period

^2^CT scanners used in less than 5% of all studies in a single hospital

### Eligibility criteria

All adult patients (≥ 18 years) were included in accordance with the BTS nodule management guideline [3]. We selected patients with at least one CT scan that fully covered the lungs (e.g., CT thorax, CT thorax-abdomen).

### Radiology report analysis

#### NLP algorithm

**Fig. 2:**
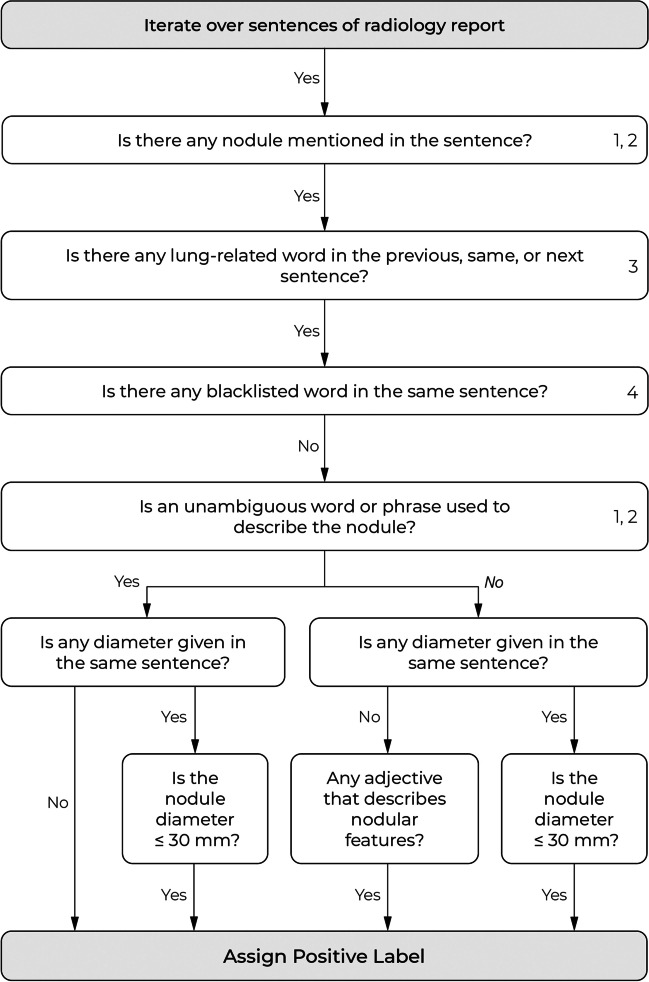
Overview of the natural language processing (NLP) algorithm for the detection of reported pulmonary nodules. The numbers on the right side of a box refer to the lookup tables in Appendix E1 (supplement)

We developed a natural language processing (NLP) algorithm to identify pulmonary nodules described in radiology reports. This algorithm is a rule-based system that uses combinations of keywords (e.g., nodule, lesion) and specific search patterns to find any reported nodule and its diameter. If one or more pulmonary nodules are detected, it returns the largest reported nodule. An overview of the algorithm is shown in Fig. 2. A detailed description of the algorithm and its development is provided in Appendix E1 (supplement).

For the development of the NLP algorithm, we created a dataset of 1000 randomly sampled radiology reports from 1000 unique patients from hospital A (*n* = 500) and hospital B (*n* = 500). These reports were annotated by W.H. and a medical student under supervision of an experienced radiologist (M.R., 26 years of experience): each report was given a label indicating (1) whether a pulmonary nodule was reported and (2) the diameter of the largest reported nodule if available (otherwise, a missing value was registered). For testing the NLP algorithm, we created an independent dataset of 200 randomly sampled radiology reports from 200 unique patients from hospital A (*n* = 100) and hospital B (*n* = 100). There was no overlap between patients from the development and test set. The test set was annotated by an experienced radiologist (E.T.S., 32 years of experience) according to the same annotation procedure as applied for the development set.

We evaluate the NLP algorithm by measuring the sensitivity and specificity for detecting reports with pulmonary nodules on the development and test set. For the subset of true positive detections, we report the sensitivity and specificity for detecting nodule diameter measurements and also report the accuracy for correctly detecting the largest reported nodule diameter.

#### Analysis and definitions

Per center, we calculated the annual number of chest CTs on study and patient level. A study was marked as “positive” if the report described at least one reported pulmonary nodule with a maximum diameter of 30 mm, regardless of nodule morphology, type (i.e., solid, part-solid, non-solid, calcified, or perifissural), and malignancy status. A study was marked as “new positive” if it was not preceded by another positive study within the last 2 years, which is generally the maximum duration between nodule follow-up examinations [3, 4]. However, this may not fully avoid duplicate counts of rare findings such as persistent subsolid nodules or stable hamartoma [10, 11].

We included the following subanalyses: First, we calculated the number of new nodule findings with a minimum diameter of 5 mm for which follow-up is recommended [3]. These lesions are clinically most relevant and should be measured and reported consistently at hospitals A and B. Second, we calculated the number of new nodule findings per CT protocol (i.e., scanner model, slice thickness). Third, the total number of patients with a new positive finding was calculated for subgroups which were stratified by age and sex. Finally, for each year, a subanalysis was conducted for patients with chest CT scans without a history of malignancy (both extrapulmonary and pulmonary) within the last 10 years. Patients with (prior) cancer are more likely to develop pulmonary nodules (i.e., metastases). Moreover, the yearly influx of non-cancer patients should be tracked in order to properly interpret the results of the lung cancer analysis (see the next section).

### Lung cancer analysis

To investigate the relationship between nodule detection and early lung cancer detection, we calculated the incidence of lung cancer diagnoses within 2 years after a new positive chest CT scan. By only taking new positive chest CT scans, duplicate nodule counts were avoided for patients with multiple follow-up examinations over the years. We focused on the trend analysis of stage I cancers, as they include detections of nodules instead of masses (> 30 mm) [12]. For this analysis, newly reported nodules were manually linked to the corresponding stage I cancer diagnoses by an experienced radiologist (E.T.S.). The details of this procedure are provided in Appendix E2 (supplement).

The total number of yearly new positive CT studies with consequent lung cancer diagnosis was calculated and then stratified by cancer stage according to the respective TNM classification at the time of diagnosis (editions 5–8) [12]. Carcinoma in situ or cancers with missing TNM staging were excluded. According to the NCR, lung cancer diagnoses were based on histological examination, cytology testing, or clinical diagnostic testing (e.g., medical imaging, exploratory surgery). An overview of the number of diagnoses per basis is provided in Appendix E3 (supplement).

## Results

### Evaluation of the NLP algorithm

The algorithm had a sensitivity of 94% (62/66) and specificity of 96% (128/134) for identifying radiology reports with pulmonary nodules in the test set. For the subset of true positive detections, it had a sensitivity of 90% (27/30) and specificity of 97% (31/32) for identifying those with nodule diameter measurements. The algorithm had an accuracy of 93% (25/27) for correctly detecting the largest reported nodule diameter. An overview of the evaluation metrics on the development and test set is provided in Table 2.

**Table 2:** Evaluation scores of the NLP algorithm on the development and test dataset

		Development dataset	Test dataset
Number of reports	All	1000	200
	Hospital A	500	100
	Hospital B	500	100
Positive labels (*n*, % of all positive labels)	All	348	66
	With nodule diameter	186 (53%)	32 (48%)
	Without nodule diameter	162 (47%)	34 (52%)
Negative labels		652	134
Detecting nodules (%, proportion)	Sensitivity	94.0 (327/348)	93.9 (62/66)
	Specificity	96.3 (628/652)	95.5 (128/134)
Detecting nodule diameter^1^ (%, proportion)	Sensitivity	92.2 (165/179)	90.0 (27/30)
	Specificity	98.0 (144/148)	96.9 (31/32)
Detecting largest nodule diameter^2^ (%, proportion)	Accuracy	97.6 (161/165)	92.6 (25/27)

^1^Only applicable to the subset of true positive reports

^2^Only applicable to the subset of true positive reports with nodule diameter measurements

### Radiology report analysis

#### Results on scan level

Between 2008 and 2019, 166,688 chest CT studies were performed in hospitals A and B (Table 1). From these studies, 98,479 chest CT studies were conducted in hospital A and 68,209 in hospital B (Table 1). The total annual number of chest CT studies more than doubled from 9955 in 2008 to 20,476 in 2019 (Table 3). The average number of chest CT scans per patient only slightly increased from 1.45 in 2008 to 1.54 in 2019.

**Table 3:** Annual number of positive chest CT scans in hospitals A and B, patient and scan-level data (2008–2019)

		Patients with positive finding (*n* (%) of total patients)			Positive studies (*n* (%) of total studies)	
Year	Total patients	Any nodule^1^	Nodule with diameter ≥ 5 mm	Total studies	Any nodule^1^	Nodule with diameter ≥ 5 mm
2008	6845	2595 (37.9)	994 (14.5)	9955	3806 (38.2)	1362 (13.7)
2009	6986	2494 (35.7)	925 (13.2)	10,019	3611 (36.0)	1294 (12.9)
2010	7477	2738 (36.6)	1017 (13.6)	10,426	3901 (37.4)	1339 (12.8)
2011	7574	2889 (38.1)	1173 (15.5)	10,613	4090 (38.5)	1552 (14.6)
2012	8117	3262 (40.2)	1307 (16.1)	11,328	4538 (40.1)	1685 (14.9)
2013	8910	3748 (42.1)	1694 (19.0)	12,567	5420 (43.1)	2311 (18.4)
2014	9711	4234 (43.6)	1969 (20.3)	13,943	6160 (44.2)	2696 (19.3)
2015	10,659	4895 (45.9)	2283 (21.4)	15,516	7218 (46.5)	3234 (20.8)
2016	10,964	5281 (48.2)	2616 (23.9)	15,823	7725 (48.8)	3667 (23.2)
2017	11,536	5849 (50.7)	3004 (26.0)	17,007	8828 (51.9)	4224 (24.8)
2018	12,689	6268 (49.4)	3032 (23.9)	19,015	9562 (50.3)	4211 (22.1)
2019	13,286	6654 (50.1)	3108 (23.4)	20,476	10,523 (51.4)	4407 (21.5)

^1^Also includes pulmonary nodules without reported diameter

During the same period, the total number of positive chest CT studies increased from 3806 (38%) in 2008 to 10,523 (51%) in 2019 (Table 3). For positive findings with a minimum nodule diameter of 5 mm, the total number of positive chest CT studies tripled from 1362 (14%) in 2008 to 4407 (22%) in 2019. The percentage of positive chest CT studies reached a plateau in 2017 and then remained constant in both hospitals. The trend analyses per hospital are included in Appendix E4 (supplement). The incidence of new positive chest CT studies per CT protocol is provided in Appendix E5 (supplement).

#### Results on patient level

**Fig. 3:**
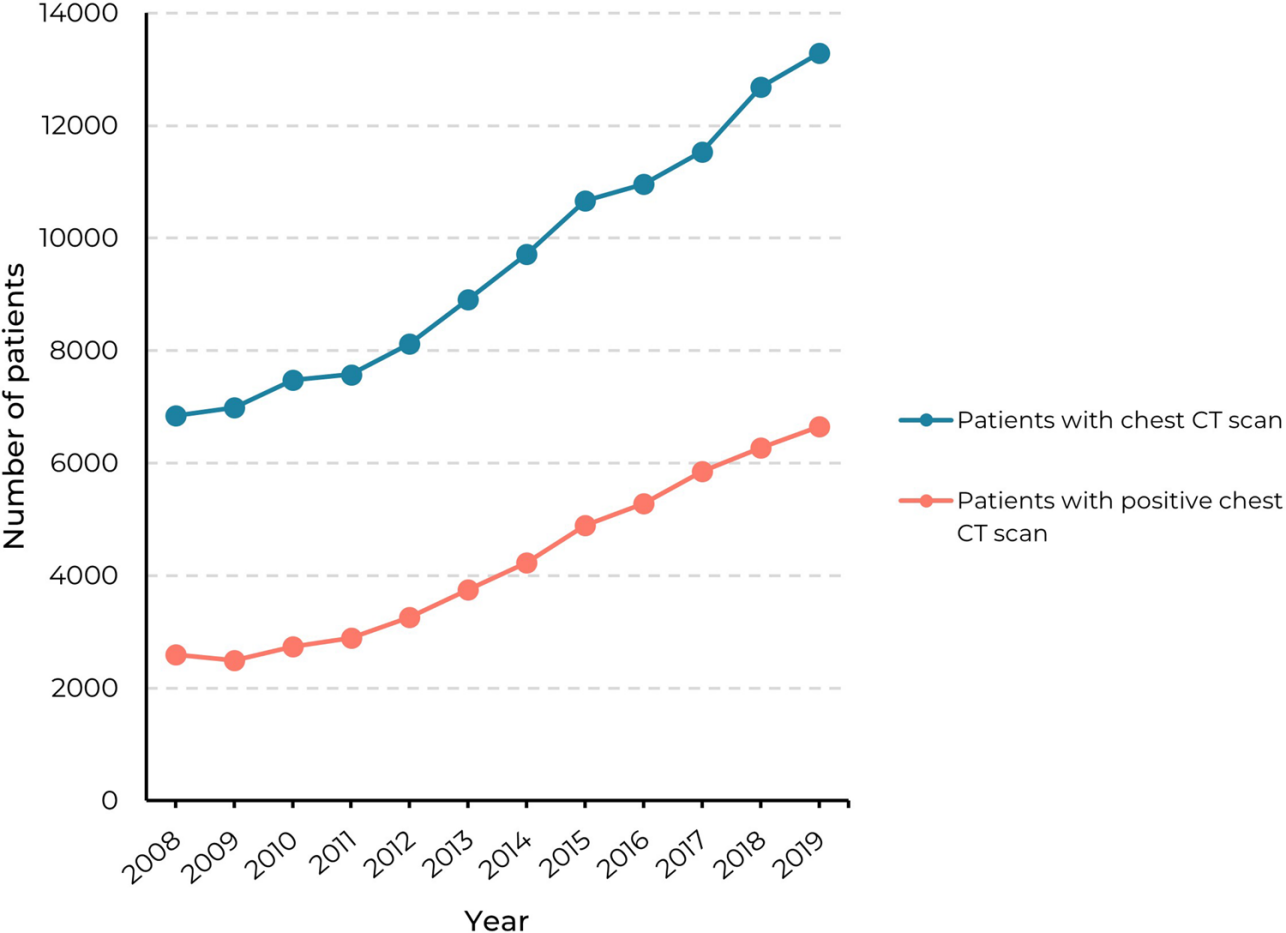
Annual number of unique patients with a (positive) chest CT scan in hospitals A and B in the period 2008–2019

In hospitals A and B, 74,803 unique patients underwent a chest CT scan between 2008 and 2019 (Table 1). From these patients, 40,440 patients were examined in hospital A and 34,363 patients in hospital B (Table 1). The total number of patients who underwent a chest CT scan doubled from 6845 in 2008 to 13,286 in 2019 (Table 3). The total number of patients with a positive finding increased from 2595 (38%) in 2008 to 6654 (50%) in 2019. In Fig. 3, this trend is visualized and compared with the total annual number of patients who underwent a chest CT scan. For positive findings with a minimum nodule diameter of 5 mm, the total number of patients with a positive finding tripled from 994 (15%) in 2008 to 3108 (23%) in 2019. The mean age of patients who underwent a chest CT scan linearly increased from 58.1 ± 15.2 years in 2008 to 62.6 ± 14.3 years in 2019.

The total annual number of patients with a new positive finding more than doubled from 2006 (out of 6954, 29%) in 2010 to 4107 (out of 11,258, 37%) in 2019 (Table 4). For new positive findings with a minimum nodule diameter of 5 mm, the total number of patients increased from 608 (9%) in 2010 to 1611 (14%) in 2019. A subanalysis of the patients without any prior cancer diagnosis can be found in Appendix E6 (supplement). The yearly percentage of this group remained constant during the investigated period (56.0 ± 1.5%). An overview of the nodule size distribution per year is included in Appendix E7 (supplement).

**Table 4:** Annual number of patients with new positive chest CT scans and those followed by lung cancer diagnosis within 2 years in hospitals A and B (2010–2019)

		Patients with new positive chest CT scan (*n*, % of total patients)^1^		Patients with new positive chest CT scan and subsequent stage I lung cancer diagnosis within 2 years (*n*, % of total patients)^1^
Year	Total patients^1^	Any nodule^2^	Nodule with diameter ≥ 5 mm	Any nodule^3^
2010	6954	2006 (28.8)	608 (8.7)	26 (0.4)
2011	7021	2100 (29.9)	743 (10.6)	30 (0.4)
2012	7570	2451 (32.4)	837 (11.1)	33 (0.4)
2013	8147	2736 (33.6)	1056 (13.0)	49 (0.6)
2014	8848	3031 (34.3)	1186 (13.4)	63 (0.7)
2015	9467	3382 (35.7)	1325 (14.0)	65 (0.7)
2016	9564	3518 (36.8)	1462 (15.3)	73 (0.8)
2017	9883	3812 (38.6)	1660 (16.8)	78 (0.8)
2018	10,890	4012 (36.8)	1613 (14.8)	NA
2019	11,258	4107 (36.5)	1611 (14.3)	NA

^1^Excluded all patients with a positive chest CT scan within the previous 2 years

^2^Also includes pulmonary nodules without reported diameter

^3^Nodule and corresponding lung cancer location were manually verified (see Appendix E2)

#### Analysis of subgroups with respect to age

**Fig. 4:**
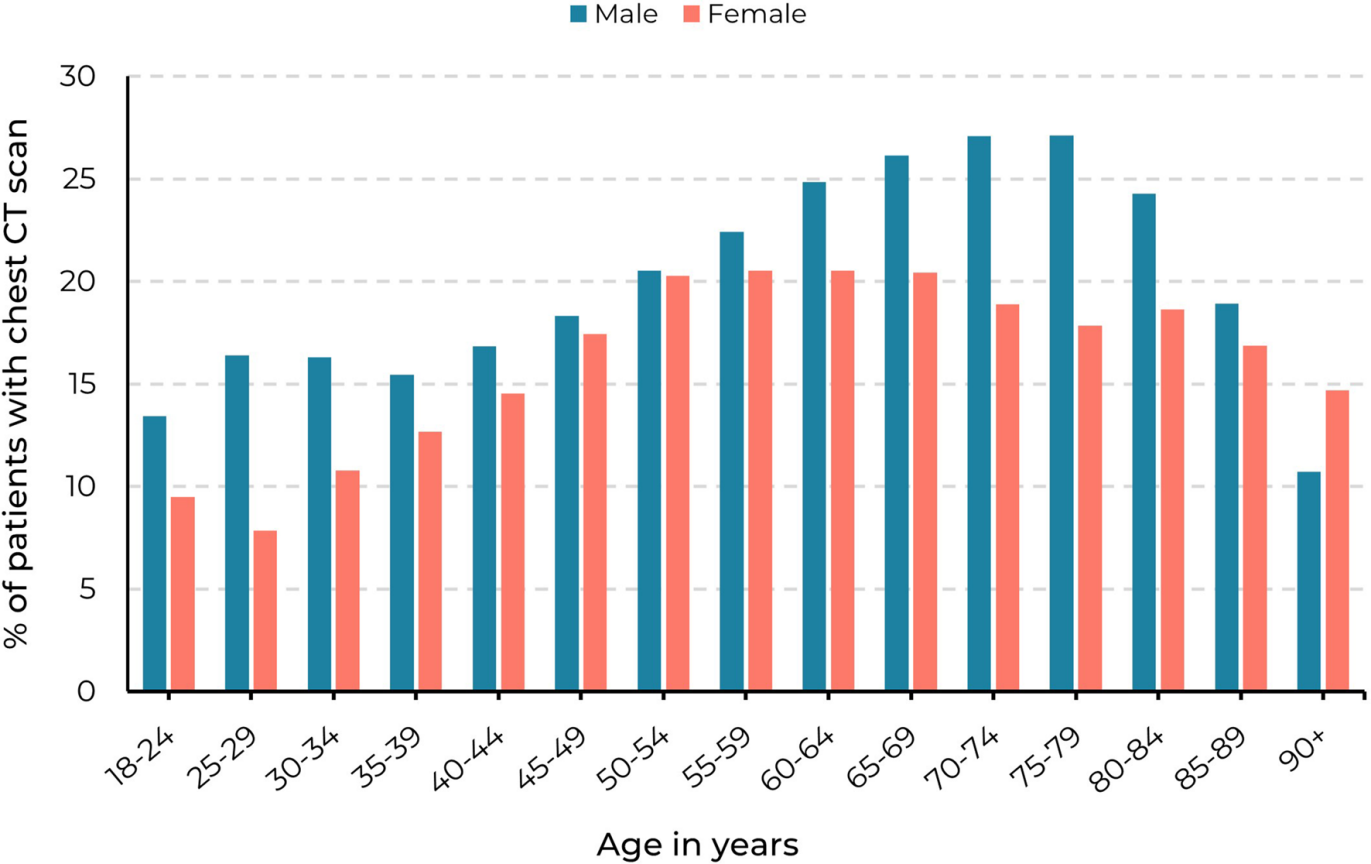
The proportion of patients with a new positive chest CT scan in hospitals A and B in the period 2010–2019, grouped by age and sex. The denominator is the number of patients with a chest CT per age group. Only the first new positive finding of a patient is counted

The incidence of new nodule findings increased with age until the age of 70 years (Fig. 4): from 486 (23%) in the age group of 18 to 24 years (*n* = 2120) to 5014 (47%) in the age group of 65 to 69 years (*n* = 10,764). After the age of 70 years, the incidence decreased: from 4514 (46%) in the age group of 70 to 74 years (*n* = 9819) to 121 (25%) in the age group of 90 years or older (*n* = 476). New nodules were more frequently reported in men in comparison to women in all age groups (16,383 men versus 12,975 women), except in the age group of 90 years or older.

### Lung cancer analysis

**Fig. 5:**
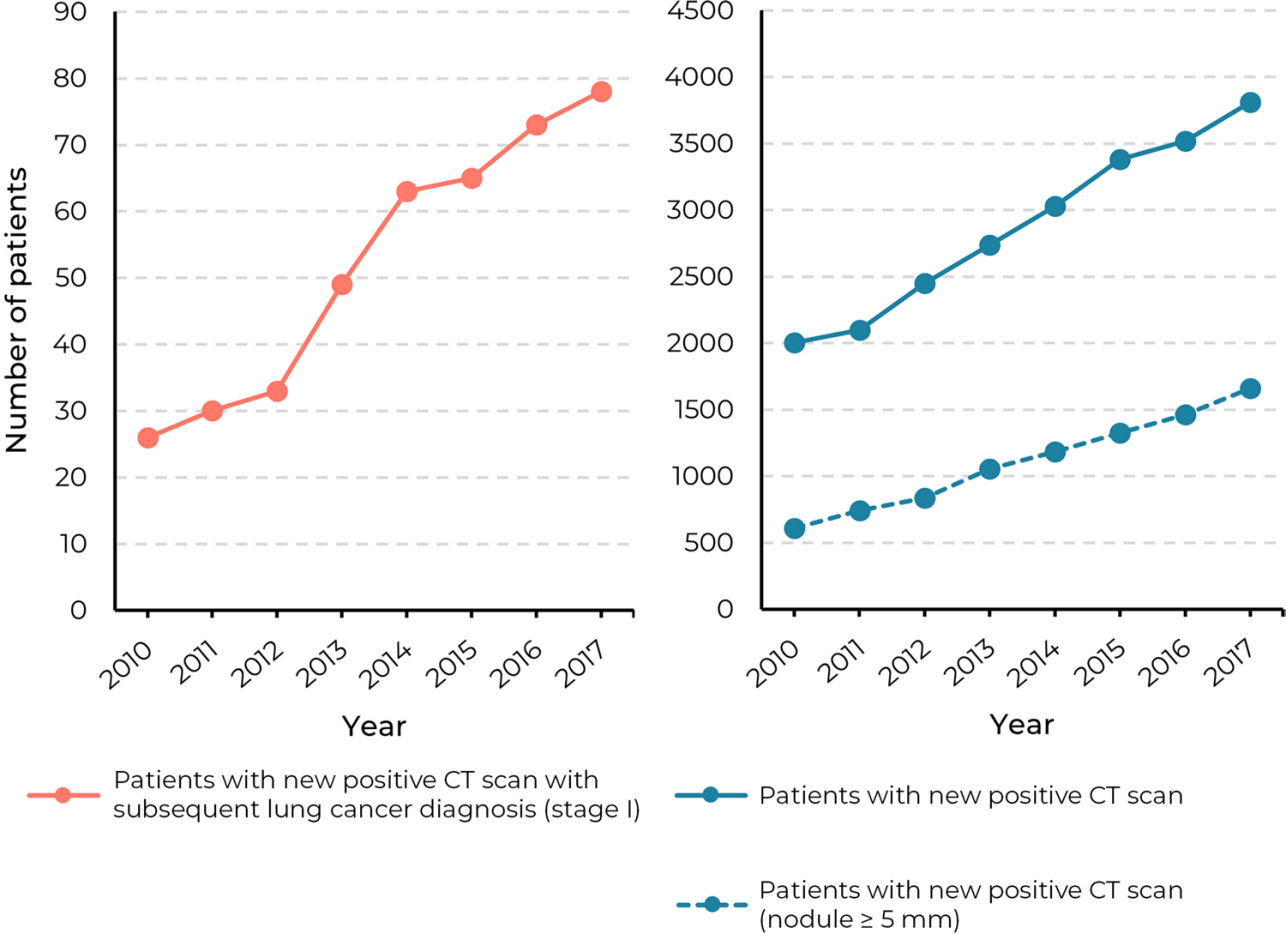
The number of patients with a new positive CT scan (all nodules and nodules  ≥ 5 mm) compared with those with a subsequent stage I lung cancer diagnosis within 2 years in hospitals A and B in the period 2010–2017

The total annual number of patients with a new positive finding with a subsequent stage I lung cancer diagnosis increased from 26 (out of 6954, 0.4%) in 2010 to 78 (out of 9883, 0.8%) in 2017 (Table 4). In Fig. 5, this trend is visualized and compared with the total annual number of patients with a new positive finding. A subanalysis of the patients without a prior cancer diagnosis can be found in Appendix E6 (supplement). The analyses for lung cancer stages II, III, and IV are included in Appendix E8 (supplement).

## Discussion

In this retrospective study, we analyzed an extensive dataset of radiology reports and lung cancer diagnoses records from two Dutch hospitals, one academic and one teaching hospital, over a period of 12 years. Radiology reports were analyzed using a validated NLP algorithm. In the period between 2008 and 2019, we found that the annual number of chest CT studies doubled and the number of positive studies almost tripled. The nodule incidence increased from 38 to 51% in all chest CT studies. The number of patients in whom new pulmonary nodules were reported also doubled in the period between 2010 and 2019. By linking our results to the Netherlands Cancer Registry, we also found that the more frequent identification of pulmonary nodules was accompanied by additional cases of stage I lung cancer diagnoses.

There are various potential causes that may explain the trends that we found. The positive results from lung cancer screening trials and the aging patient population have probably contributed to the more frequent identification of pulmonary nodules. Results from large lung cancer screening trials such as NLST [2] and NELSON [1] were published during the investigated period and likely raised awareness among radiologists for the potential risks of pulmonary nodules and the need of monitoring for early lung cancer detection. Another explanation could be the aging patient population, considering that the nodule incidence increases with age (until the age of 70 years), as supported by our analysis.

It is uncertain whether improvements in the quality of the performed chest CT scans contributed to the increased nodule incidence: the 16- and 64-slice CT scanners were gradually phased out and replaced by newer 128-slice, 160-slice, and 320-slice CT scanners at both hospitals between 2011 and 2017. At hospital A, we found a higher nodule incidence for CT scans with low slice thickness (< 1 mm) from the newer scanners compared to scans with a higher slice thickness (≥ 3 mm) from the older scanners (Appendix E5, supplement). At hospital B, we conversely found a higher nodule incidence in scans with a high slice thickness (≥ 3 mm) from all scanners. Therefore, advances in CT technology may have contributed in hospital A, but probably not in hospital B.

We found that the trends in the annual number of CT studies and (new) positive CT studies were largely similar between hospitals A and B. It is noticeable that the percentage of positive studies reached a plateau in 2017 in both hospitals. A possible explanation is the publication of the BTS guidelines in 2015 [3], which could have resulted in fewer reported small (≤ 5 mm) or clearly benign nodules. Furthermore, the updated Fleischner nodule management guidelines were later published in 2017, in which the minimal threshold size for follow-up of solitary solid nodules was increased from 4 to 6 mm [4]. When comparing the overall nodule incidence between the hospitals, the nodule incidence was substantially higher in hospital A as compared to that in hospital B (44% vs. 29% in 2008, 59% vs. 41% in 2019). This may be explained by the fact that hospital A is an academic hospital, where pulmonary nodules are more researched. Furthermore, the impact of institution (academic versus non-academic) as well as CT technology is most likely influenced by the individual readers’ performance [13].

Our findings correspond to those from Gould et al [6] who reported increases in the identification of pulmonary nodules in chest CT more than a decade ago. They found that pulmonary nodules with a minimum diameter of 4 mm were identified in 29% of all scans performed between 2006 and 2012. For any reported nodule, our estimate is 45% for all scans performed between 2008 and 2019 and 19% when using a nodule diameter threshold of 5 mm. A key difference in our findings is that Gould et al found that the increases in nodule detection did not identify additional cases of lung cancer, whereas we found that increased nodule identifications were associated with more stage I lung cancer diagnoses within 2 years. The latter might be among other factors attributable to the results of the NLST trial in 2011 [2] that has led to increased knowledge but also higher awareness of the importance of small nodules for the detection of stage I cancers.

This study has several limitations. First, our NLP algorithm takes all pulmonary nodules into consideration and cannot rule out nodules that were described with typical benign features (e.g., calcified or perifissural nodules or micronodules). An increase of these nodules would not lead to additional CT follow-up [3, 4]. However, the diameter of these low-risk nodules is typically not measured and therefore, these lesions were largely ignored in our subanalyses where a diameter threshold of 5 mm was applied. Second, an automated analysis of free-text radiology reports may not give the most accurate estimation of the incidence of pulmonary nodules due to inter- and intra-observer variability among reading radiologists. A more accurate method could be an automated analysis of the CT scans with AI systems, although this would require a substantial CT database, a clinically validated AI system, and a significant amount of computing resources. Finally, CT scans that only contain portions of the lungs (e.g., CT scan of the abdomen, neck, or heart) were not included in the analysis. However, the analysis of chest CT scans should already include the vast majority of the reported incidental pulmonary nodules in clinical practice.

In conclusion, we observed that the number of patients who underwent chest CT examinations substantially increased over the past decade, as did the number of patients in whom a pulmonary nodule was identified. The more frequent pulmonary nodule identifications were associated with more stage I lung cancer diagnoses. The results need to be validated in a larger, geographically diverse cohort. However, these preliminary findings suggest that the more frequent chest CT scans and incidental nodule identifications lead to an increased detection of early-stage lung cancer, and stress the importance of efficient nodule management.

### Supplementary Information

Below is the link to the electronic supplementary material.

Supplementary file1 (PDF 433 KB)

## Abbreviations

AI: Artificial intelligence
BTS: British Thoracic Society
EHR: Electronic Health Record
IKNL: Netherlands Comprehensive Cancer Organization
NCR: Netherlands Cancer Registry
NLP: Natural language processing
PACS: Picture Archiving and Communication System
TNM: TNM Classification of Malignant Tumours

## Declarations

### Guarantor

The scientific guarantor of this publication is Colin Jacobs.

### Statistics and biometry

No complex statistical methods were necessary for this paper.

### Methodology

• Retrospective

• Observational

• Multicenter study
