# Supplementary material for: Trends in the incidence of pulmonary nodules in chest computed tomography: 10-year results from two Dutch hospitals
Source: Eur Radiol. 2023 Jun 20;33(11):8279–88. doi: 10.1007/s00330-023-09826-3 (PMC10598118; doi:10.1007/s00330-023-09826-3)
Supplement: Supplementary file 1 — Supplementary file1 (PDF 433 KB) [file 330_2023_9826_MOESM1_ESM.pdf]

Trends in the Incidence of Pulmonary Nodules in Chest Computed Tomography: 10-Year Results from Two Dutch Hospitals

ELECTRONIC SUPPLEMENTARY MATERIAL

References

1. Mikolov T, Chen K, Corrado G, Dean J (2013) Efficient estimation of word representations in vector space. DOI: 10.48550/arXiv.1301.3781
2. World Health Organization (2014) International classification of diseases for oncology ICD-O, 3rd edition, 1st revision. World Health Organization, Geneva. Available via https://apps.who.int/iris/bitstream/handle/10665/96612/9789241548496_eng.pdf Accessed 1 Aug 2022

Appendix E1 - NLP algorithm

An NLP algorithm was developed to automatically identify pulmonary nodules that are mentioned in radiology reports.

Pre-processing

Each report is pre-processed by lowercasing all words, removing letter accents, splitting punctuation and digits from words, and splitting the report into sentences. After pre-processing, the algorithm processes each report sentence by sentence.

Nodule detection

The algorithm uses keywords to (partially) match pulmonary nodules. Table E1 - E4 contain all keywords that were used to identify reported pulmonary nodules. The keywords in each table are categorized by sub keywords and keywords that are matched as a whole. In order to find all nodule synonyms, we first generated word vectors with the algorithm Word2Vec [1] by using all available radiology reports as input. Then we manually inspected the 200 nearest neighbours for the vector belonging to "nodule" to make a list of positive keywords (Table E1 and E2). Subsequently, the algorithm was iteratively updated with new rules to minimize the number of false positives on the development set (see the Materials and methods section in the main article).

In order to increase the precision of the algorithm, any nodule keyword was combined with another lung-related keyword (Table E3) in the previous, same, or next sentence. If a blacklisted keyword (Table E4) was used in the same sentence as a nodule keyword, then the nodule was ignored. Another method to increase the precision of the algorithm was to categorize nodule keywords into unambiguous and ambiguous keywords. Unambiguous keywords are synonyms of a nodule, while ambiguous keywords may describe a nodule only in specific contexts. Therefore, ambiguous keywords are combined with diameter measurements or adjectives that indicate a small or nodular shape: pointy (Dutch: "puntvormig"), spherical (Dutch: "bolvormig"), nodular (Dutch: "nodulair"), or small (Dutch: "kleine"). Any nodule description preceded by the word "no" (Dutch: "geen") was ignored, which indicates that a negation was used.

Nodule diameter detection

For determining the nodule diameter, regular expressions were used to find any valid combination of a digit and metric (mm or cm) in the same sentence as the detected pulmonary nodule. A diameter measurement was ignored if it was preceded by the words "was" or "previously"; those indicate that the measurement originates from a previous study.

Word2Vec training procedure

We trained a Word2Vec algorithm [1] with the continuous bag-of-words (CBOW) architecture on all 166,688 radiology reports in our corpus to find nodule keywords for the NLP algorithm.

We developed a custom Word2Vec implementation in PyTorch, a Python Deep Learning library. The model was trained with a Nvidia TITAN xp GPU (12 GB).

The input dataset was pre-processed by removing letter accents, lowercasing words, and tokenizing the texts. Punctuation marks were kept in the dataset. All infrequent words in the corpus (fewer than 5 occurrences) were replaced by a placeholder token in order to prune the word vocabulary (36K words). A context window of 10 words was used to generate samples, 5 words before and after the target word. Any sentence with fewer than 11 words was padded with a placeholder token. Subsequently, the dataset (24M samples) was randomly split into a training and validation set: 90% of the generated samples were used for training and the remaining 10% for validation.

The model was trained until its performance on the validation set stopped improving (this occurred after two epochs). The batch size was set to 1024 and the learning rate of the Adam optimizer was set to 0.001. The dimensionality of the word vectors was set to 128. The subsampling threshold was set to 0.001, so that higher-frequency words were randomly downsampled.

Table E1. Unambiguous keywords for finding nodules. Keywords with an asterisk can return partial matches.

Sub keywords	Translation	Keywords	Translation
haard	*lesion*	nodi	*nodules*
laesie / leasie / lesie / lasie	*lesion*	longnodi	*pulmonary nodules*
lesion	*lesion*	spn	*solitary pulmonary nodule*
nodule	*nodule*		
noduli	*nodule*		
nodulus	*nodule*		
nodus	*nodule*		
granulo*	*granuloma*		
hamarto*	*hamartoma*		
verdichtinkje	*small opacity*		
densiteitje	*small opacity*		

Table E2. Ambiguous keywords for finding nodules.

Sub keywords	Translation	Keywords	Translation
tumor	*tumour*	rip	*tumour*
longca	*lung carcinoma*	meta	*metastasis*
longkanker	*lung cancer*	afw	*abnormality*
maligniteit	*malignancy*	afwijking	*abnormality*
metastas	*metastasis*	afwijkingen	*abnormalities*
pancoast	*pancoast tumour*	opacity	*opacity*
massa	*mass*	opaciteit	*opacity*
longmeta	*lung metastases*	verandering	*change or abnormality*
proces	*tumour*	matglasafwijking	*ground glass abnormality*
densiteit	*density*	groundglassafwijking	*ground glass abnormality*
ggo	*ground glass opacity*	matglasgebied	*ground glass area*
matglas	*ground glass area*	longafwijking	*pulmonary abnormality*

Table E3. Keywords related to the lungs or bronchi. Keywords with an asterisk can return partial matches.

Sub keywords	Translation	Keywords	Translation
bovenkwab / bovenveld	*upper lobe*	lbk	*left upper lobe*
bronch*	*bronchus*	lok	*left lower lobe*
fissur / fissuur	*fissure*	mk	*middle lobe*
lingula	*lingula*	pulm*	*pulmonary*
long	*lung*	rbk	*right upper lobe*
middenkwab / middenveld	*middle lobe*	rok	*right lower lobe*
onderkwab / onderveld	*lower lobe*	apex	*apex*
pleura	*pleura*		
pulmona / pulmones	*pulmonary*		
thorax	*thorax*		
atelecta*	*atelectasis*		
hilus	*hilus*		
paracardiaal	*paracardial*		
parahilair	*parahilar*		

Table E4. Blacklisted keywords. Keywords with an asterisk can be matched partially.

Sub keywords	Translation	Keywords	Translation
wervel	*vertebra*	nieren	*kidneys*
bijnier	*adrenal*	nier	*kidney*
mamma	*breast*	blaas	*bladder*
lever	*liver*	milt	*spleen*
mediastin*	*mediastinum*	buik	*abdomen*
carina	*carina*	maag	*stomach*
retroperiton	*retroperitone*	tree-in-bud/ tree in bud	*tree-in-bud*
ossale	*ossal*	hals	*neck*
schildklier	*thyroid*	bot	*bone*
centrilobulair / centrolobulair	*centrilobular*	mesencefalon	*mesencephalon*
lytisch	*lytic*	gluteus	*gluteal*
sclerotisch	*sclerotic*	costa	*costa*
lymfo* / lymfa*	*lymphatic*	thoraxfoto	*x-thorax*
muscu*	*muscle*	station	*station*
skelet	*skeleton*	corpus	*corpus*
struma	*goitre*	cardiaal	*cardiac*
thoraxwand	*chest wall*		
cardio	*cardio*		
pancreas	*pancreas*		
coronair	*coronary*		
cerebra	*cerebra*		
contusie	*contusion*		
rectum	*rectum*		
plug*	*plugging*		
tepel	*nipple*		

borst	*breast*	
vraagstelling	*(clinical) question*	
weke delen / weke-delen / wekedelen	*soft tissue*	
x-thorax	*x-thorax*	

Appendix E2 – Nodule malignancy verification for lung cancer stage I

We manually verified the malignancy of newly reported pulmonary nodules in patients with a subsequent lung cancer stage I diagnosis within two years.

Procedure

In the period of 2010-2017, 479 new positive studies were identified with a subsequent stage I lung cancer diagnosis. For each case, all corresponding radiology reports were collected and inspected by an experienced radiologist (E.T.S.). The reported nodules were linked to the lung cancer diagnoses based on the size and lobe location as recorded in the Netherlands Cancer Registry (NCR). Any described morphology (e.g., spiculated, lobulation, etc.) or reported growth was checked as well. If the information in the radiology reports was inconclusive, then the corresponding CT scans were also assessed. Any discrepancies between the automated analysis and manual check were recorded.

Results

From the 479 new positive studies, 417 studies (87%) had a nodule that matched with a subsequent stage I lung cancer diagnosis. Table E16 shows an overview of the 62 discrepancies that were found (13%). Most cases (n=53) concerned a matched mass instead of a nodule. All matched nodular lung cancers were found to be at least 5 mm in size.

Table E5. Discrepancies between manual and automated analysis of reported nodules with subsequent stage I lung cancer diagnosis in 479 new positive studies.

Discrepancies	No. new positive studies
Match with mass, but nodules present[1]	34
Match with solitary mass, no nodules present	19
No nodules present	7
No match, but nodules present	2

[1] The nodules can be benign or can represent a metastasis or lung cancer other than stage I.

Appendix E3 - Basis of diagnosis

Table E6. Overview of the number of lung cancer diagnoses of patients who underwent a chest CT in hospitals A and B between 2000 and 2019, stratified by year and basis. Lung cancer diagnoses were selected based on the ICD-O codes C340-C349 [2] with any morphology.

Year	Total diagnoses	Histological examination (n (%) of total diagnoses)	Clinical diagnostic testing (n (%) of total diagnoses)[1]	Cytology testing (n (%) of total diagnoses)[2]	Biochemical /immunological testing (n (%) of total diagnoses)[3]
**2000**	57	48 (84.2)	5 (8.8)	4 (7.0)	0 (0.0)
**2001**	98	87 (88.8)	2 (2.0)	9 (9.2)	0 (0.0)
**2002**	112	81 (72.3)	9 (8.0)	22 (19.6)	0 (0.0)
**2003**	238	184 (77.3)	13 (5.5)	41 (17.2)	0 (0.0)
**2004**	310	261 (84.2)	19 (6.1)	30 (9.7)	0 (0.0)
**2005**	354	283 (79.9)	18 (5.1)	53 (15.0)	0 (0.0)
**2006**	390	312 (80.0)	19 (4.9)	59 (15.1)	0 (0.0)
**2007**	403	313 (77.7)	23 (5.7)	67 (16.6)	0 (0.0)
**2008**	413	295 (71.4)	34 (8.2)	84 (20.3)	0 (0.0)
**2009**	441	317 (71.9)	26 (5.9)	98 (22.2)	0 (0.0)
**2010**	438	321 (73.3)	26 (5.9)	91 (20.8)	0 (0.0)
**2011**	502	325 (64.7)	40 (8.0)	137 (27.3)	0 (0.0)
**2012**	518	355 (68.5)	36 (6.9)	127 (24.5)	0 (0.0)
**2013**	515	342 (66.4)	57 (11.1)	115 (22.3)	1 (0.2)
**2014**	493	328 (66.5)	40 (8.1)	124 (25.2)	1 (0.2)
**2015**	610	396 (64.9)	61 (10.0)	153 (25.1)	0 (0.0)
**2016**	578	353 (61.1)	77 (13.3)	148 (25.6)	0 (0.0)
**2017**	589	402 (68.3)	75 (12.7)	112 (19.0)	0 (0.0)
**2018**	685	490 (71.5)	78 (11.4)	117 (17.1)	0 (0.0)
**2019**	662	458 (69.2)	69 (10.4)	135 (20.4)	0 (0.0)
**Total**	8,406	5,951 (70.8)	727 (8.6)	1,726 (20.5)	2 (0.0)

[1] For example: medical imaging, exploratory surgery or autopsy (without confirmation by microscopy).
[2] For example: bone marrow aspiration, blood testing, or any diagnosis confirmed by microscopy, but unclear whether it concerns cytology or histology.
[3] For example: liquid biopsy testing.

Table E7. Overview of the number of extrapulmonary cancer diagnoses of patients who underwent a chest CT in hospitals A and B between 2000 and 2019, stratified by year and basis. Together with the lung cancer diagnoses, they were used to determine the history of malignancy for each patient.

Year	Total diagnoses	Histological examination (n (%) of total diagnoses)	Clinical diagnostic testing (n (%) of total diagnoses)[1]	Hematology/ cytology testing (n (%) of total diagnoses)[2]	Biochemical/ immunological testing (n (%) of total diagnoses)[3]
**2000**	895	871 (97.3)	7 (0.8)	17 (1.9)	0 (0.0)
**2001**	1,030	993 (96.4)	12 (1.2)	25 (2.4)	0 (0.0)
**2002**	1,182	1,155 (97.7)	5 (0.4)	22 (1.9)	0 (0.0)
**2003**	1,591	1,527 (96.0)	26 (1.6)	35 (2.2)	3 (0.2)
**2004**	1,755	1,677 (95.6)	28 (1.6)	48 (2.7)	2 (0.1)
**2005**	1,924	1,848 (96.0)	24 (1.2)	48 (2.5)	4 (0.2)
**2006**	2,297	2,197 (95.6)	36 (1.6)	62 (2.7)	2 (0.1)
**2007**	2,431	2,324 (95.6)	38 (1.6)	66 (2.7)	3 (0.1)
**2008**	2,480	2,354 (94.9)	57 (2.3)	68 (2.7)	1 (0.0)
**2009**	2,778	2,617 (94.2)	66 (2.4)	93 (3.3)	2 (0.1)
**2010**	2,823	2,663 (94.3)	49 (1.7)	106 (3.8)	5 (0.2)
**2011**	2,992	2,844 (95.1)	47 (1.6)	97 (3.2)	4 (0.1)
**2012**	3,233	3,069 (94.9)	59 (1.8)	101 (3.1)	4 (0.1)
**2013**	3,247	3,039 (93.6)	101 (3.1)	103 (3.2)	4 (0.1)
**2014**	3,283	3,117 (94.9)	68 (2.1)	93 (2.8)	5 (0.2)
**2015**	3,214	3,056 (95.1)	75 (2.3)	79 (2.5)	4 (0.1)
**2016**	3,737	3,548 (94.9)	105 (2.8)	77 (2.1)	7 (0.2)
**2017**	3,974	3,780 (95.1)	104 (2.6)	79 (2.0)	11 (0.3)
**2018**	4,088	3,871 (94.7)	125 (3.1)	79 (1.9)	13 (0.3)
**2019**	3,794	3,551 (93.6)	153 (4.0)	79 (2.1)	11 (0.3)
**Total**	52,748	50,101 (95.0)	1,185 (2.2)	1,377 (2.6)	85 (0.2)

[1] For example: medical imaging, exploratory surgery or autopsy (without confirmation by microscopy).
[2] For example: bone marrow aspiration, blood testing, or any diagnosis confirmed by microscopy, but unclear whether it concerns cytology or histology.
[3] For example: liquid biopsy testing.

Appendix E4 - Hospital-specific analyses

Hospital A

Table E8. Annual number of positive chest CT scans in hospital A, patient and scan-level data (2008-2019).

		Patients with positive finding (n (%) of total patients)			Positive studies (n (%) of total studies)	
**Year**	**Total patients**	**Any nodule[1]**	**Nodule with diameter ≥ 5 mm**	**Total studies**	**Any nodule[1]**	**Nodule with diameter ≥ 5 mm**
**2008**	4,172	1,813 (43.5)	693 (16.6)	6,414	2,794 (43.6)	997 (15.5)
**2009**	4,068	1,713 (42.1)	647 (15.9)	6,053	2,564 (42.4)	936 (15.5)
**2010**	4,168	1,832 (44.0)	703 (16.9)	6,001	2,708 (45.1)	955 (15.9)
**2011**	4,232	1,902 (44.9)	802 (19.0)	6,178	2,755 (44.6)	1,086 (17.6)
**2012**	4,531	2,118 (46.7)	910 (20.1)	6,599	3,052 (46.2)	1,202 (18.2)
**2013**	4,838	2,424 (50.1)	1,102 (22.8)	7,253	3,638 (50.2)	1,547 (21.3)
**2014**	5,156	2,679 (52.0)	1,245 (24.1)	7,930	4,056 (51.1)	1,760 (22.2)
**2015**	5,747	3,184 (55.4)	1,484 (25.8)	8,851	4,837 (54.6)	2,167 (24.5)
**2016**	6,268	3,519 (56.1)	1,695 (27.0)	9,502	5,277 (55.5)	2,441 (25.7)
**2017**	6,582	3,851 (58.5)	1,811 (27.5)	10,157	5,931 (58.4)	2,520 (24.8)
**2018**	7,108	3,974 (55.9)	1,798 (25.3)	11,336	6,344 (56.0)	2,474 (21.8)
**2019**	7,387	4,308 (58.3)	1,799 (24.4)	12,205	7,169 (58.7)	2,509 (20.6)

[1] Also includes pulmonary nodules without reported diameter.

Table E9. Annual number of patients with new positive CT scan and those followed by lung cancer diagnosis within two years in hospital A (2010-2019).

		Patients with new positive chest CT scan (n, % of total patients)[1]		Patients with new positive chest CT scan and subsequent stage I lung cancer diagnosis within two years (n, % of total patients)[1]
**Year**	**Total patients[1]**	**Any nodule[2]**	**Nodule with diameter ≥ 5 mm**	**Any nodule[3]**
**2010**	3,738	1,269 (33.9)	390 (10.4)	14 (0.4)
**2011**	3,801	1,302 (34.3)	478 (12.6)	12 (0.3)
**2012**	4,129	1,542 (37.3)	554 (13.4)	15 (0.4)
**2013**	4,319	1,711 (39.6)	648 (15.0)	23 (0.5)
**2014**	4,591	1,863 (40.6)	701 (15.3)	23 (0.5)
**2015**	4,949	2,165 (43.7)	834 (16.9)	25 (0.5)
**2016**	5,332	2,308 (43.3)	915 (17.2)	38 (0.7)
**2017**	5,450	2,453 (45.0)	953 (17.5)	36 (0.7)
**2018**	5,882	2,432 (41.3)	904 (15.4)	NA
**2019**	6,002	2,558 (42.6)	899 (15.0)	NA

[1] Excluded all patients with a positive chest CT scan within the previous 2 years.

[2] Also includes pulmonary nodules without reported diameter.

[3] Nodule and corresponding lung cancer location were manually verified (see Appendix E2).

Table E10. Annual number of patients with new positive chest CT scans followed by lung cancer diagnosis in hospital A (2010-2017), stratified by cancer stage (II-IV) according to the respective TNM Classification at the time of diagnosis.

		Patients with new positive chest CT scan and subsequent lung cancer diagnosis within two years (n, % of total patients)[1]					
		Cancer stage II		Cancer stage III		Cancer stage IV[3]	
**Year**	**Total patients[1]**	**Any nodule[2]**	**Nodule with diameter ≥ 5 mm**	**Any nodule[2]**	**Nodule with diameter ≥ 5 mm**	**Any nodule[2]**	**Nodule with diameter ≥ 5 mm**
**2010**	3,738	9 (0.2)	2 (0.1)	13 (0.3)	7 (0.2)	27 (0.7)	13 (0.3)
**2011**	3,801	9 (0.2)	8 (0.2)	18 (0.5)	13 (0.3)	34 (0.9)	13 (0.3)
**2012**	4,129	15 (0.4)	8 (0.2)	19 (0.5)	11 (0.3)	38 (0.9)	22 (0.5)
**2013**	4,319	7 (0.2)	5 (0.1)	21 (0.5)	11 (0.3)	31 (0.7)	18 (0.4)
**2014**	4,591	9 (0.2)	4 (0.1)	16 (0.3)	14 (0.3)	24 (0.5)	15 (0.3)
**2015**	4,949	11 (0.2)	3 (0.1)	19 (0.4)	11 (0.2)	39 (0.8)	29 (0.6)
**2016**	5,332	12 (0.2)	9 (0.2)	36 (0.7)	25 (0.5)	32 (0.6)	21 (0.4)
**2017**	5,450	14 (0.3)	8 (0.1)	19 (0.3)	13 (0.2)	31 (0.6)	22 (0.4)

[1] Excluded all patients with a positive chest CT scan within the previous 2 years.

[2] Also includes pulmonary nodules without reported diameter.

[3] It should be noted that pulmonary nodules (e.g., metastases) are counted in patients with pulmonary masses.

Hospital B

Table E11. Annual number of positive chest CT scans in hospitals B, patient and scan-level data (2008-2019).

		Patients with positive finding (n (%) of total patients)			Positive studies (n (%) of total studies)	
**Year**	**Total patients**	**Any nodule[1]**	**Nodule with diameter ≥ 5 mm**	**Total studies**	**Any nodule[1]**	**Nodule with diameter ≥ 5 mm**
**2008**	2,673	782 (29.3)	301 (11.3)	3,541	1,012 (28.6)	365 (10.3)
**2009**	2,918	781 (26.8)	278 (9.5)	3,966	1,047 (26.4)	358 (9.0)
**2010**	3,309	906 (27.4)	314 (9.5)	4,425	1,193 (27.0)	384 (8.7)
**2011**	3,342	987 (29.5)	371 (11.1)	4,435	1,335 (30.1)	466 (10.5)
**2012**	3,586	1,144 (31.9)	397 (11.1)	4,729	1,486 (31.4)	483 (10.2)
**2013**	4,072	1,324 (32.5)	592 (14.5)	5,314	1,782 (33.5)	764 (14.4)
**2014**	4,555	1,555 (34.1)	724 (15.9)	6,013	2,104 (35.0)	936 (15.6)
**2015**	4,912	1,711 (34.8)	799 (16.3)	6,665	2,381 (35.7)	1,067 (16.0)
**2016**	4,696	1,762 (37.5)	921 (19.6)	6,321	2,448 (38.7)	1,226 (19.4)
**2017**	4,954	1,998 (40.3)	1,193 (24.1)	6,850	2,897 (42.3)	1,704 (24.9)
**2018**	5,581	2,294 (41.1)	1,234 (22.1)	7,679	3,218 (41.9)	1,737 (22.6)
**2019**	5,899	2,346 (39.8)	1,309 (22.2)	8,271	3,354 (40.6)	1,898 (22.9)

[1] Also includes pulmonary nodules without reported diameter.

Table E12. Annual number of patients with new positive chest CT scans and those followed by lung cancer diagnosis within two years in hospital B (2010-2019).

		Patients with new positive chest CT scan (n, % of total patients)[1]		Patients with new positive chest CT scan and subsequent stage I lung cancer diagnosis within two years (n, % of total patients)[1]
**Year**	**Total patients[1]**	**Any nodule[2]**	**Nodule with diameter ≥ 5 mm**	**Any nodule[3]**
**2010**	3,216	737 (22.9)	218 (6.8)	12 (0.4)
**2011**	3,220	798 (24.8)	265 (8.2)	18 (0.6)
**2012**	3,441	909 (26.4)	283 (8.2)	18 (0.5)
**2013**	3,828	1,025 (26.8)	408 (10.7)	26 (0.7)
**2014**	4,257	1,168 (27.4)	485 (11.4)	40 (0.9)
**2015**	4,518	1,217 (26.9)	491 (10.9)	40 (0.9)
**2016**	4,232	1,210 (28.6)	547 (12.9)	35 (0.8)
**2017**	4,433	1,359 (30.7)	707 (15.9)	42 (0.9)
**2018**	5,008	1,580 (31.5)	709 (14.2)	NA
**2019**	5,256	1,549 (29.5)	712 (13.5)	NA

[1] Excluded all patients with a positive chest CT scan within the previous 2 years.

[2] Also includes pulmonary nodules without reported diameter.

[3] Nodule and corresponding lung cancer location were manually verified (see Appendix E2).

Table E13. Annual number of patients with new positive chest CT scans followed by lung cancer diagnosis in hospital B (2010-2017), stratified by cancer stage (II-IV) according to the respective TNM Classification at the time of diagnosis.

		**Patients with new positive chest CT scan and subsequent lung cancer diagnosis within two years (n, % of total patients)[1]**					
		**Cancer stage II**		**Cancer stage III**		**Cancer stage IV[3]**	
**Year**	**Total patients[1]**	**Any nodule[2]**	**Nodule with diameter ≥ 5 mm**	**Any nodule[2]**	**Nodule with diameter ≥ 5 mm**	**Any nodule[2]**	**Nodule with diameter ≥ 5 mm**
**2010**	3,216	7 (0.2)	3 (0.1)	28 (0.9)	14 (0.4)	57 (1.8)	18 (0.6)
**2011**	3,220	5 (0.2)	2 (0.1)	29 (0.9)	15 (0.5)	52 (1.6)	21 (0.7)
**2012**	3,441	11 (0.3)	6 (0.2)	25 (0.7)	12 (0.3)	77 (2.2)	34 (1.0)
**2013**	3,828	14 (0.4)	5 (0.1)	28 (0.7)	16 (0.4)	73 (1.9)	47 (1.2)
**2014**	4,257	6 (0.1)	5 (0.1)	35 (0.8)	18 (0.4)	73 (1.7)	47 (1.1)
**2015**	4,518	12 (0.3)	7 (0.2)	39 (0.9)	26 (0.6)	59 (1.3)	30 (0.7)
**2016**	4,232	8 (0.2)	4 (0.1)	22 (0.5)	15 (0.4)	67 (1.6)	41 (1.0)
**2017**	4,433	20 (0.5)	10 (0.2)	42 (0.9)	31 (0.7)	71 (1.6)	51 (1.2)

[1] Excluded all patients with a positive chest CT scan within the previous 2 years.

[2] Also includes pulmonary nodules without reported diameter.

[3] It should be noted that pulmonary nodules (e.g., metastases) are counted in patients with pulmonary masses.

Appendix E5 – CT protocol analysis

Table E14. The number of CT studies per CT protocol in hospital A in the period of 2010-2019 (59,848 studies in total).

Slice thickness range in mm[1]	CT scanner	Number of studies (n, % of total studies)[2]	Number of new positive studies (n, % of protocol specific studies)[2]	Years active[3]
0-1	SIEMENS Sensation 64	1,474 (2.5%)	694 (47.1%)	2011-2016
	Canon Aquilion One	7,972 (13.3%)	1,979 (24.8%)	2012-2019
	Canon Aquilion CXL	1,771 (3.0%)	776 (43.8%)	2016-2019
	Canon Aquilion Precision	4,353 (7.3%)	1,792 (41.2%)	2017-2019
	**Subtotal**	**15,570 (26.0%)**	**5,241 (33.7%)**	
1-2	SIEMENS Sensation 64	5,049 (8.4%)	1,533 (30.4%)	2011-2016
	SIEMENS Sensation 16	5,932 (9.9%)	1,875 (31.6%)	2010-2016
	Canon Aquilion One	14,568 (24.3%)	4,396 (30.2%)	2012-2019
	SIEMENS Biograph 40	2,339 (3.9%)	1,088 (46.5%)	2012-2019
	Canon Aquilion CXL	4,257 (7.1%)	1,478 (34.7%)	2016-2019
	Canon Aquilion Precision	1,503 (2.5%)	582 (38.7%)	2017-2018
	**Subtotal**	**33,648 (56.2%)**	**10,952 (32.5%)**	
2-3	SIEMENS Sensation 16	4,451 (7.4%)	1,373 (30.8%)	2011-2016
3-4	SIEMENS Sensation 16	3,635 (6.1%)	1,017 (28.0%)	2010-2011
N/A	Other[4]	2,544 (4.3%)	1,020 (40.1%)	2010-2015

[1] Based on the CT reconstruction with the most slices. The reconstruction with the lowest slice thickness is selected in case of multiple suitable reconstructions. The upper limit of the provided slice thickness range is exclusive.

[2] Excluded all chest CT scans with a positive scan within the previous 2 years in order to prevent duplicate counts.

[3] These years account for 95% of all scans made with the specified CT protocol.

[4] CT protocols used in less than 5% of all studies.

Table E15. The number of CT studies per CT protocol in hospital B in the period of 2010-2019 (50,056 studies in total).

Slice thickness range in mm[1]	CT scanner	Number of studies (n, % of total studies)[2]	Number of new positive studies (n, % of total studies)[2]	Years active[3]
0-1	SIEMENS Sensation 64	5,064 (10.1%)	603 (11.9%)	2010,2012-2018
	SIEMENS SOMATOM Definition Flash	1,201 (2.4%)	111 (9.2%)	2012-2019
	SIEMENS SOMATOM Definition AS+	498 (1.0%)	60 (12.0%)	2018-2019
	**Subtotal**	**6,763 (13.5%)**	**774 (11.4%)**	
1-2	SIEMENS Sensation 64	3,762 (7.5%)	695 (18.5%)	2010-2017
	SIEMENS SOMATOM Definition Flash	2,365 (4.7%)	571 (24.1%)	2011-2019
	SIEMENS SOMATOM Definition AS+	1,597 (3.2%)	302 (18.9%)	2017-2019
	**Subtotal**	**7,724 (15.4%)**	**1,568 (20.3%)**	
2-3	SIEMENS Sensation 64	2,444 (4.9%)	287 (11.7%)	2010-2017
	SIEMENS SOMATOM Definition AS+	590 (1.2%)	66 (11.2%)	2017-2019
	**Subtotal**	**3,034 (6.1%)**	**353 (11.6%)**	
3-4	SIEMENS Sensation 64	13,151 (26.3%)	3,703 (28.2%)	2010-2018
	SIEMENS SOMATOM Definition Flash	15,392 (30.7%)	4,232 (27.5%)	2012-2019
	SIEMENS SOMATOM Definition AS+	643 (1.3%)	171 (26.6%)	2017-2019
	SIEMENS SOMATOM Definition Edge	1,162 (2.3%)	304 (26.2%)	2019
	**Subtotal**	**30,348 (60.6%)**	**8,410 (27.7%)**	
NA	Other[4]	2,187 (4.4%)	447 (20.4%)	2010-2019

[1] Based on the CT reconstruction with the most slices. The reconstruction with the lowest slice thickness is selected in case of multiple suitable reconstructions. The upper limit of the provided slice thickness range is exclusive.

[2] Excluded all chest CT scans with a positive scan within the previous 2 years in order to prevent duplicate counts.

[3] These years account for 95% of all scans made with the specified CT protocol.

[4] CT protocols used in less than 5% of all studies.

Appendix E6 - Stage I lung cancer analysis for patients without history of malignancy

Table E16. Annual number of patients with new positive chest CT scans and those followed by lung cancer diagnosis within two years in hospitals A and B (2010-2019). All patients did not have any history of malignancy (both extrapulmonary and pulmonary) within 10 years before the CT examination.

		Patients with new positive chest CT scan (n, % of total patients)[1]		Patients with new positive chest CT scan with no cancer diagnosis within two years (n, % of total patients)[1]		Patients with new positive chest CT scan and subsequent stage I lung cancer diagnosis within two years (n, % of total patients)[1]
**Year**	**Total patients[1]**	**Any nodule[2]**	**Nodule with diameter ≥ 5 mm**	**Any nodule[2]**	**Nodule with diameter ≥ 5 mm**	**Any nodule[3]**
**2010**	4,013	937 (23.3)	257 (6.4)	682 (17.0)	153 (3.8)	15 (0.4)
**2011**	3,931	955 (24.3)	318 (8.1)	694 (17.7)	194 (4.9)	22 (0.6)
**2012**	4,089	1,128 (27.6)	361 (8.8)	839 (20.5)	236 (5.8)	24 (0.6)
**2013**	4,463	1,259 (28.2)	498 (11.2)	935 (21.0)	330 (7.4)	30 (0.7)
**2014**	4,823	1,450 (30.1)	591 (12.3)	1,106 (23.1)	404 (8.4)	43 (0.9)
**2015**	5,452	1,721 (31.6)	703 (12.9)	1,282 (23.9)	475 (8.8)	43 (0.8)
**2016**	5,523	1,777 (32.2)	748 (13.5)	1,337 (24.5)	511 (9.4)	46 (0.8)
**2017**	5,637	1,940 (34.4)	900 (16.0)	1,461 (26.2)	619 (11.1)	49 (0.9)
**2018**	6,114	1,970 (32.2)	823 (13.5)	NA	NA	NA
**2019**	6,155	1,930 (31.4)	810 (13.2)	NA	NA	NA

[1] Excluded all patients with a positive chest CT scan within the previous 2 years.
[2] Also includes pulmonary nodules without reported diameter.
[3] Nodule and corresponding lung cancer location were manually verified (see Appendix E2).

Appendix E7 - Pulmonary nodule diameter distribution

Table E17. Diameter distribution of newly reported pulmonary nodules per year, hospital A and B combined (2010-2019). Only the largest reported pulmonary nodule finding per study is counted, which has not been preceded by another pulmonary nodule finding within the previous two years.

		Nodule diameter in mm (n, % of total nodules)					
**year**	**Total**	**< 5**	**≥ 5 to < 6**	**≥ 6 to < 8**	**≥ 8 to < 15**	**> 15**	**Not reported**
**2010**	2,006	103 (5.1)	72 (3.6)	104 (5.2)	196 (9.8)	236 (11.8)	1,295 (64.6)
**2011**	2,100	89 (4.2)	82 (3.9)	116 (5.5)	239 (11.4)	306 (14.6)	1,268 (60.4)
**2012**	2,451	160 (6.5)	87 (3.5)	150 (6.1)	270 (11.0)	330 (13.5)	1,454 (59.3)
**2013**	2,736	199 (7.3)	163 (6.0)	199 (7.3)	364 (13.3)	330 (12.1)	1,481 (54.1)
**2014**	3,031	293 (9.7)	168 (5.5)	244 (8.1)	395 (13.0)	379 (12.5)	1,552 (51.2)
**2015**	3,382	392 (11.6)	209 (6.2)	273 (8.1)	433 (12.8)	410 (12.1)	1,665 (49.2)
**2016**	3,518	524 (14.9)	214 (6.1)	335 (9.5)	496 (14.1)	417 (11.9)	1,532 (43.5)
**2017**	3,812	540 (14.2)	265 (7.0)	390 (10.2)	560 (14.7)	445 (11.7)	1,612 (42.3)
**2018**	4,012	519 (12.9)	237 (5.9)	386 (9.6)	518 (12.9)	472 (11.8)	1,880 (46.9)
**2019**	4,107	463 (11.3)	224 (5.5)	342 (8.3)	590 (14.4)	455 (11.1)	2,033 (49.5)
**All**	31,155	3282 (10.5)	1721 (5.5)	2539 (8.1)	4061 (13.0)	3780 (12.1)	15,772 (50.6)

Appendix E8 - Annual number of patients with new positive chest CT scans followed by lung cancer diagnosis stage II-IV

Table E18. Annual number of patients with new positive chest CT scans followed by lung cancer diagnosis in hospitals A and B (2010-2017), stratified by cancer stage (II-IV) according to the respective TNM Classification at the time of diagnosis.

		Patients with positive chest CT scan and subsequent lung cancer diagnosis within two years (n, % of total patients)[1]					
		Cancer stage II		Cancer stage III		Cancer stage IV[3]	
**Year**	**Total patients[1]**	**Any nodule[2]**	**Nodule with diameter ≥ 5 mm**	**Any nodule[2]**	**Nodule with diameter ≥ 5 mm**	**Any nodule[2]**	**Nodule with diameter ≥ 5 mm**
**2010**	6,954	16 (0.2)	5 (0.1)	41 (0.6)	21 (0.3)	84 (1.2)	31 (0.4)
**2011**	7,021	14 (0.2)	10 (0.1)	47 (0.7)	28 (0.4)	86 (1.2)	34 (0.5)
**2012**	7,570	26 (0.3)	14 (0.2)	44 (0.6)	23 (0.3)	115 (1.5)	56 (0.7)
**2013**	8,147	21 (0.3)	10 (0.1)	49 (0.6)	27 (0.3)	104 (1.3)	65 (0.8)
**2014**	8,848	15 (0.2)	9 (0.1)	51 (0.6)	32 (0.4)	97 (1.1)	62 (0.7)
**2015**	9,467	23 (0.2)	10 (0.1)	58 (0.6)	37 (0.4)	98 (1.0)	59 (0.6)
**2016**	9,564	20 (0.2)	13 (0.1)	58 (0.6)	40 (0.4)	100 (1.0)	62 (0.6)
**2017**	9,883	34 (0.3)	19 (0.2)	61 (0.6)	44 (0.4)	102 (1.0)	73 (0.7)

[1] Excluded all patients with a positive chest CT scan within the previous 2 years.

[2] Also includes pulmonary nodules without reported diameter.

[3] It should be noted that pulmonary nodules (e.g., metastases) are counted in patients with pulmonary masses.
